# Concentration vs. Optical Density of ESKAPEE Bacteria: A Method to Determine the Optimum Measurement Wavelength

**DOI:** 10.3390/s24248160

**Published:** 2024-12-21

**Authors:** Bruno Wacogne, Marine Belinger Podevin, Naïs Vaccari, Claudia Koubevi, Céline Codjiová, Emilie Gutierrez, Lucie Davoine, Marjorie Robert-Nicoud, Alain Rouleau, Annie Frelet-Barrand

**Affiliations:** 1 Institut FEMTO-ST, Université de Franche-Comté, CNRS, F-25000 Besançon, France; marine.podevin@hotmail.com (M.B.P.); nais.vaccari@hotmail.com (N.V.); claudiakoubevi@yahoo.fr (C.K.); codjiovaceline@gmail.com (C.C.); emilie.gutierrez@enil-mamirolle.fr (E.G.); luciedavoine25@gmail.com (L.D.); alain.rouleau@femto-st.fr (A.R.); annie.frelet-barrand@femto-st.fr (A.F.-B.); 2 Centre d’Investigation Clinique, Centre Hospitalier Universitaire de Besançon, INSERM CIC 1431, 25030 Besançon, France; 3 Smaltis, Bioinnovation, Rue Charles Bried, 25030 Besançon, France; marjorie.robertnicoud@smaltis.fr

**Keywords:** ESKAPEE bacteria, optical density, white light spectroscopy, absorbance spectra, spectra processing

## Abstract

Optical density measurement has been used for decades to determine the microorganism concentration and more rarely for mammalian cells. Although this measurement can be carried out at any wavelength, studies report a limited number of measurement wavelengths, mainly around 600 nm, and no consensus seems to be emerging to propose an objective method for determining the optimum measurement wavelength for each microorganism. In this article, we propose a method for analyzing the absorbance spectra of ESKAPEE bacteria and determining the optimum measurement wavelength for each of them. The method is based on the analysis of the signal-to-noise ratio of the relationships between concentrations and optical densities when the measurement wavelength varies over the entire spectral range of the absorbance spectra measured for each bacterium. These optimum wavelengths range from 612 nm for *Enterococcus faecium* to 705 nm for *Acinetobacter baumannii*. The method can be directly applied to any bacteria, any culture method, and also to any biochemical substance with an absorbance spectrum without any particular feature such as an identified maximum.

## 1. Introduction

Optical density (OD), a concentration measurement method based on the linear Beer-Lambert relationship between particle concentration and light absorbance, is a widely used method in biology, particularly for estimating bacterial growth and enumeration (the non-linearity of OD versus concentration at high concentrations is not considered here). The origins of the technique can be traced back to advances in spectroscopy in the early 20th century, when light absorbance was first used to measure the concentration of solutes in a solution [1]. In bacterial studies, optical density at specific wavelengths (usually 600 nm, referred to as OD600) became a standard way of quantifying cultures, although spectral measurement enriched bacteria characterization [2]. The underlying principle to choose a measurement wavelength is that light is scattered and absorbed as it passes through a bacterial suspension. As bacteria concentration increases, more light is scattered, resulting in higher OD values [3,4]. OD measurement provides a rapid and non-destructive estimate of bacterial growth, which is particularly useful in routine microbiology laboratories for experiments requiring real-time growth monitoring. However, OD readings do not directly correspond to viable bacteria counts and require calibration with colony-forming unit (CFU) counts to convert optical measurements into relevant/meaningful biological data [5]. Despite this limitation, OD measurement remains a cornerstone of bacterial enumeration in fields ranging from basic research to industry.

Typically, OD measurements are performed at a specific wavelength, most commonly 600 nm (OD600), although other wavelengths such as 595 nm, 540 nm, and 650 nm are sometimes used depending on the experimental setup. These wavelengths are chosen primarily based on the need to avoid interference from cellular components and medium and to maximize the scattering properties of bacteria. OD600 is the most commonly used wavelength due to its effectiveness in detecting bacteria densities in liquid cultures. Bacteria such as *Escherichia coli*, *Bacillus subtilis*, and *Pseudomonas aeruginosa* are typically measured at this wavelength. The reason for this choice relies on the properties of visible light and bacteria. Bacteria scatter light most efficiently at wavelengths around 600 nm [6]. However, measurements between 600 nm and 700 nm can be used [7]. The main advantage of OD600 is that bacterial suspensions scatter light and do not significantly absorb light at this wavelength [2,7]. In addition, many bacterial growth media, such as Luria–Bertani (LB) or nutrient broth, exhibit minimal absorbance at 600 nm (the yellowish color of culture medium indicating a strong absorption around 430–480 nm), which reduces background noise and improves the accuracy of bacterial concentration measurements.

While OD600 is the most commonly used, other wavelengths such as 595 nm and 540 nm are used in specific cases. OD590 has been used with *Staphylococcus aureus*, *Salmonella typhimurium*, or *Escherichia coli* to study the antimicrobial activity of silver nanoparticles [8]. OD measurements at several wavelengths (from 500 nm to 860 nm) have been considered for studying purple phototropic bacteria, and OD590 was preferred for experimental stability reasons [9]. OD540 has been used to estimate bacterial growth rates from turbidimetric and viable count experiments [5]. Another alternative is the use of higher wavelengths, which reduce the sensitivity to light scattering from bacteria, allowing more accurate measurements in highly concentrated cultures. OD650 was used to reveal the potential of rice straw as a substrate for biosurfactant production by hydrocarbonoclastic bacteria [10]. OD750 was employed in a study investigating the use of monocultures and a microalgae consortium to remove nitrogen and phosphate from acid casein factory effluent [11]. It was also used to measure algal growth as it avoids light absorption by cellular pigments (chlorophyll and carotenoids) [12].

The examples of wavelengths used to measure optical densities are almost endless. The choice of wavelength is sometimes influenced by the need to avoid interference from the culture medium. Most standard growth media, such as LB medium, do not absorb significantly at 600 nm, making OD600 an ideal choice. However, when using media with components that absorb light in the visible spectrum (e.g., pigments, complex carbohydrates), alternative wavelengths may be selected to ensure that the measurement reflects bacterial turbidity rather than medium interference [7]. Bacteria scatter light rather than absorb it. The efficiency of light scattering depends on the size and refractive index of cells [13]. Since bacteria are typically 0.5 to 5 μm in diameter, they scatter light most effectively in the visible spectrum. The choice of wavelengths at which ODs are measured is rarely explained in the literature. The main reasons given are that bacteria diffract light and absorption is minimal [3,7]. However, no method based on objective criteria has yet been proposed to determine the bacterial optimal measurement wavelength, e.g., in terms of potential measurement accuracy. Since most spectrophotometers and plate readers are designed to operate optimally in the visible light range (400–700 nm), it is very likely that an optimum wavelength can be determined within this spectral range for each microorganism studied.

In this article, we propose a method to determine the optimal measurement wavelength for each bacterium of the ESKAPEE group [14]. ESKAPEE is an acronym referring to a model group of bacteria involved in nosocomial diseases: *Escherichia coli*, *Staphylococcus aureus*, *Klebsiella pneumoniae*, *Acinetobacter baumannii*, *Pseudomonas aeruginosa*, *Enterococcus faecium*, and *Enterobacter cloacae*. After presenting the materials and methods section, the relationships between concentration and DO measured at 600 nm are presented for each species. These relationships are then generalized to all wavelengths contained in the measured absorbance spectra. This makes it possible to determine the optimum measurement wavelength for each bacterium by analyzing the signal-to-noise ratio of the DO concentration relationships. A discussion is suggested before concluding.

## 2. Materials and Methods

In this paper we used the spectra recorded in a previous study on the shape description of the absorption spectra of ESKAPEE bacteria. All bacteria stored at −80 °C were thawed on TSA gelose and incubated overnight. Precultures were made with 4 different clones in 10 mL of medium (LB, except TSB was used for *E. cloacae*) overnight at 225 rpm. They were centrifuged at 7180× *g* for 10 min at RT, and pellets were resuspended in an appropriate volume of PBS to adjust to an optical density of 1 ± 0.05 at 600 nm using a spectrophotometer (Biowave DNA, Biochrom Ltd., Cambridge, UK). The bacterial suspensions (10 mL) were then centrifuged at 9000× *g* for 15 min at RT, and the pellets were resuspended in 1 mL of PBS. The complete process of bacterial preparation, enumeration, and experimental setup for recording transmission spectra can be found in [4].

A total of 255 spectra were treated. For each bacterium, 5 different dilution ranges were prepared. Table 1 summarizes the number of data points for each bacterium. A data point is defined as a recorded transmittance spectrum together with the corresponding bacterial enumeration. The following equivalence was used: 1 bact.mL^−1^ = 1 cfu.mL^−1^. Table 1 also shows the concentration ranges for each bacterium.

Transmittance spectra were transformed into optical densities (OD = absorbance) spectra using conventional transformations [13]. Linear regressions of the OD vs. bacteria concentrations were calculated using the curve fitting toolbox of Matlab^TM^ R2020b software (Matlab^TM^, USA, supplier France). Noise was extracted by subtracting the smoothed experimental curve from the initial slightly noisy experimental curve. Smoothing was performed using the “spline smoothing function” of the Matlab^TM^ curve fitting toolbox with a smoothing parameter equal to 2.36 × 10^−5^. Noise envelopes were determined using the Matlab^TM^ “envelope” function with the ‘rms’ option and a window of 50 samples.

## 3. Results

This section describes a method to determine the optimum wavelength to which OD measurements should be performed for each ESKAPEE bacterium. First, OD spectra of all bacteria were used to extract bacteria’s ‘OD vs. concentration’ relationships considering a measurement wavelength of 600 nm according to that most commonly used for microorganism enumeration. Second, these relationships were calculated for each bacterium for measurement wavelengths varying over the whole spectral range. This allowed calculating the bacteria concentrations measured for a certain OD value for any measurement wavelength. Third, these results were used to define the optimum measurement wavelength for each bacterium. In what follows, ODvC stands for ‘OD vs. Concentration’ for conciseness.

### 3.1. ODvC Relationships at 600 nm

**Figure 1:**
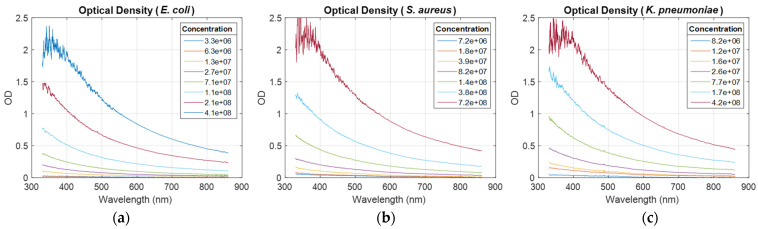
Examples of OD spectra recorded for (**a**) *E. coli*, (**b**) *S. aureus*, and (**c**) *K. pneumoniae*. Legends report concentration for each spectrum.

OD spectra were recorded for all ESKAPEE bacteria (illustration for *E. coli*, *S. aureus*, and *K. pneumoniae*, Figure 1). The light scattering and its 1/λ behavior for bacteria-sized particles were clearly visible. Spectra measured at high concentration were noisy in the short wavelength range. This will be discussed in Section 4.

**Figure 2:**
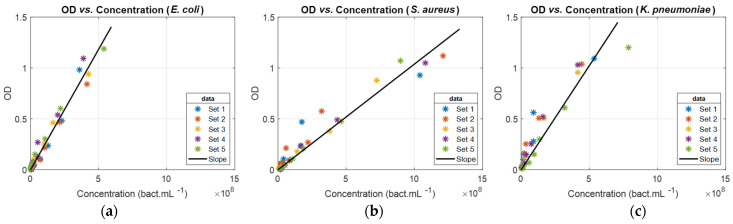
Examples of OD vs. concentration for (**a**) *E. coli*, (**b**) *S. aureus*, and (**c**) *K. pneumoniae*. ODs were measured from spectra in Figure 1 at 600 nm wavelength.

Considering spectral data measured at a 600 nm wavelength, ODs were plotted as a function of bacteria concentrations. The result for the same bacteria as Figure 1, together with linear regressions, was obtained by fitting experimental data with Equation (1) (Figure 2).

(1) $${OD}_{600}={Slope}_{600}\times Concentration$$

Bacteria concentrations in the abscissa of Figure 2 were measured using the common enumeration method described in [4]. They are indeed the concentrations reported in this reference. The ODs in ordinate of Figure 2 were measured from absorption spectra recorded in reference [4] and transformed into OD spectra (examples in Figure 1).

The ODvC relationship was linear for all bacteria in the considered concentration ranges. Slopes differ from one bacterium to another depending on the intrinsic absorption and light scattering properties related to their respective sizes. The slopes obtained at 600 nm wavelength and bacteria concentration corresponding to OD_600_ = 0.5 were calculated (Table 2).

### 3.2. Wavelength Dependences of ODvC Relationships

**Figure 3:**
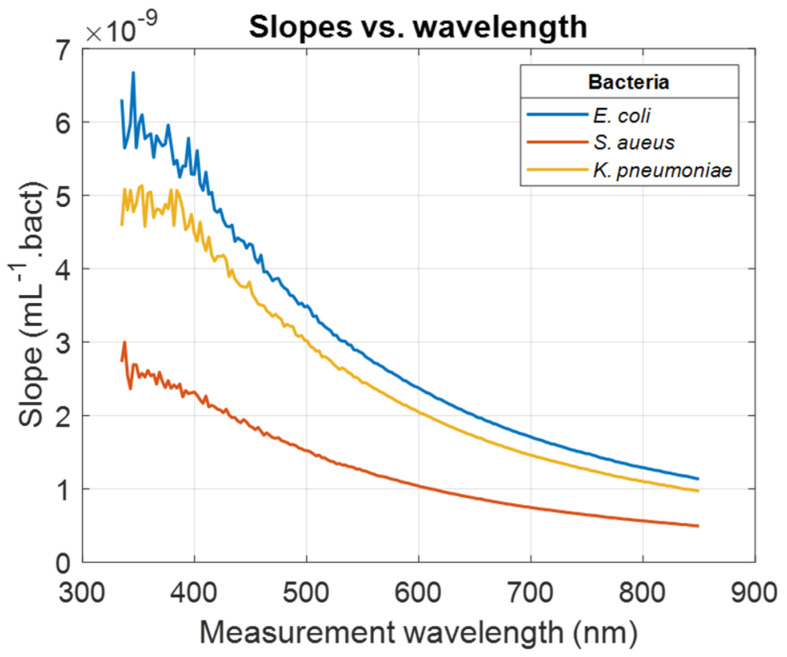
Examples of wavelength-dependent slopes for *E. coli*, *S. aureus*, and *K. pneumoniae*.

**Figure 4:**
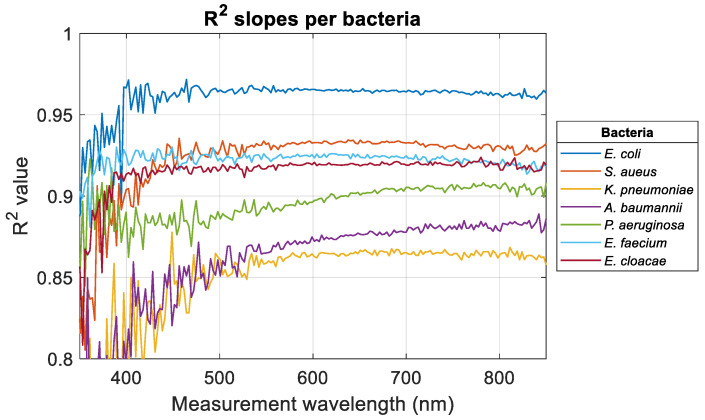
Wavelength-dependent R^2^ values obtained when fitting data with Equation (1).

Results in Section 3.1 were obtained from spectra illustrated in Figure 1 considering a measurement wavelength of 600 nm. The same calculations were conducted considering every wavelength in the spectral measurement range. Wavelength-dependent evolutions of the slope of the ODvC relationships were calculated (illustration for *E. coli*, *S. aureus*, and *K. pneumoniae*, Figure 3). Wavelength-dependent evolutions of the R^2^ obtained when fitting data with Equation (1) were calculated for all bacteria (Figure 4).

For measurement wavelengths less than 400 nm, slopes and R^2^ values were noisy for reasons that will be discussed in Section 4. Except for *A. baumannii* and *P. aeruginosa*, R^2^ values tended to stabilize when the measurement wavelength increased. For *K. pneumoniae*, R^2^ started to stabilize for a much larger measurement wavelength than other bacteria. In any case, the high R^2^ values indicated that the ODvC relationships were linear for any measurement wavelength. Table 3 shows the wavelengths for which R^2^ stabilized (when it did) and the average R^2^ value in the stabilized range (or R^2^ range when no stabilization was observed).

At this stage, it is concluded that the more or less linear aspect of the relationships was therefore not a criterion for determining the optimum measurement wavelength.

### 3.3. A Method to Determine the Optimum OD Measurement Wavelength

Evolutions of the concentrations with measurement wavelengths for a certain OD value were calculated according to Equation (2),

(2) $$C_{Bact.}\left(\lambda \right)=\frac{OD}{{Slope}_{Bact.}(\lambda )}$$

**Figure 5:**
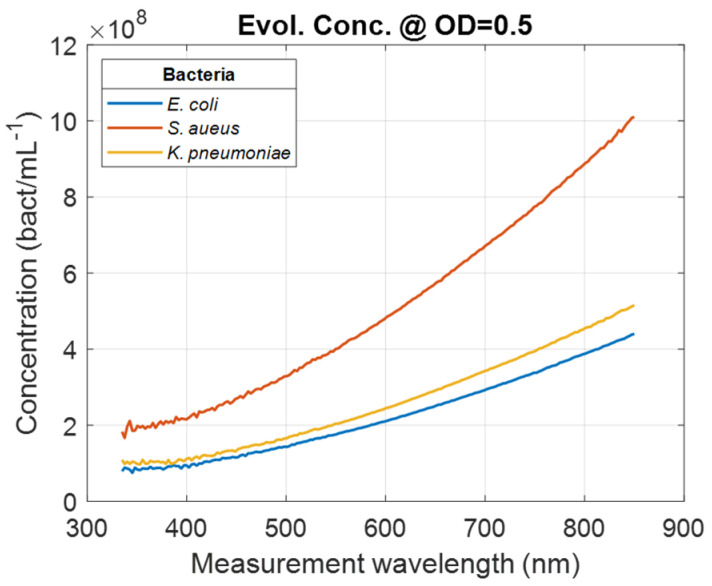
Examples of wavelength-dependent bacteria concentrations for *E. coli*, *S. aureus*, and *K. pneumoniae* calculated at OD = 0.5.

In Equation (2), $C_{Bact.}\left(\lambda \right)$ and ${Slope}_{Bact.}(\lambda )$ represent the concentration and the slope at wavelength *λ* for bacteria *Bact.*, respectively. Wavelength-dependent evolutions of the bacteria concentration calculated for OD = 0.5 was calculated for all bacteria (illustration *for E. coli*, *S. aureus*, *and K. pneumoniae*, Figure 5).

**Figure 6:**
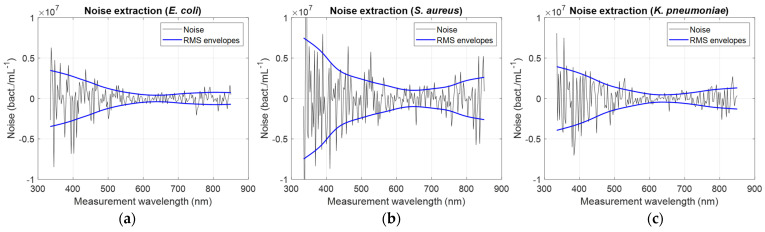
Examples of noise extraction for (**a**) *E. coli*, (**b**) *S. aureus*, and (**c**) *K. pneumoniae*. Black curve: extracted noise. Blue curves: ‘rms’ envelopes.

A way of determining the optimum OD measurement wavelength is to study the evolution of the signal-to-noise ratio (SNR) as a function of the measurement wavelength. Noise was extracted from the “concentration/measurement wavelength” relationships. Relationships were smoothed using the spline smoothing function in Matlab^TM^. Smoothed relationships were then subtracted from raw data to extract the noise. This was performed for all bacteria (illustration for *E. coli*, *S. aureus*, and *K. pneumoniae*, as well as corresponding ‘rms’ noise envelopes, Figure 6).

Noise amplitude reached minimum values between 600 and 700 nm wavelength, depending on bacteria. Considerations about the noise will be discussed in Section 4. Table 4 (first two rows) shows the minimum values of the noise amplitudes as well as the wavelengths for which these minima are reached.

**Figure 7:**
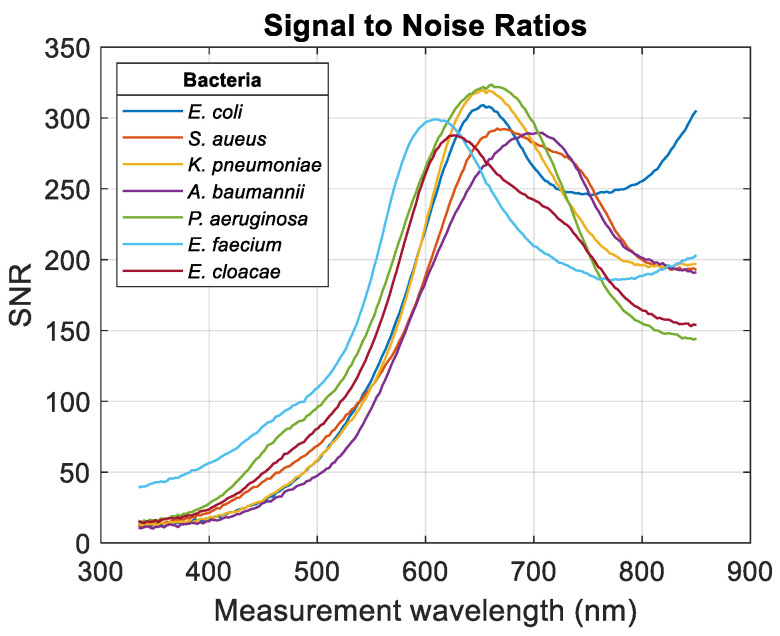
Signal to noise ratios obtained for ESKAPEE bacteria.

However, the important value was not the wavelengths for which the noise amplitudes were minimum but the wavelengths for which the signal (examples in Figure 5) to noise (examples in Figure 6) ratios (SNRs) were obtained. SNRs were then calculated depending on the measurement wavelength (Figure 7).

**Figure 8:**
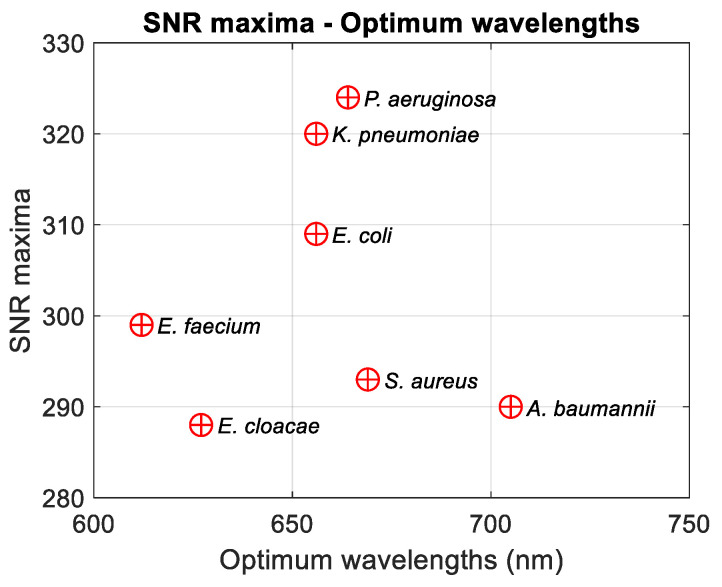
Optimum measurement wavelengths and corresponding SNRs for ESKAPEE bacteria.

SNRs reached maxima values at different wavelengths depending on bacteria. This constituted a criterion for determining the optimum measurement wavelengths. Increases in the SNR for *E. coli* and *E. faecium* are due to the almost stationary noise envelopes at large wavelengths. This will be discussed in Section 4. The optimal wavelengths are contained within a 100 nm wide range, which may appear relatively small. To date, we have no explanation for this. Maximum SNRs and corresponding wavelengths were calculated (Figure 8). Numerical values are summarized in Table 4 (rows 3 and 4). It should be noted that there is no correlation between optimal measurement wavelengths and the bio-physical characteristics of bacteria (Gram+/−, shell/bacillus, or dimensions).

The SNR varied a lot over the spectral range, hence the potential measurement accuracy. For measurement wavelengths equal to the minimum wavelength in Table 3, the measurement accuracy was low, while it reached a maximum for the optimal measurement wavelength. The measurement accuracy can be assessed through the inverse of the SNR value. For *E. coli*, for example, the accuracy at the minimum R^2^ stabilization wavelength is 100/SNR (400 nm) = 100/17 = 6%. The accuracy reaches 100/309 = 0.32% for the SNR maximum value. The accuracy variation ranges are shown in Table 4 (last row).

It should be noted that the accuracies obtained at the optimum measurement wavelength result only from the calculated SNR. It does not include inaccuracies due to difficulties in handling bacteria and/or expertise in performing bacterial enumerations.

These results showed that by measuring the evolution of SNR with the measurement wavelength, it was possible to determine the optimal wavelength at which OD could be measured. To the best of our knowledge, this is the first time that an objective method based on optical spectral data analysis has been proposed for this purpose.

## 4. Discussion

### 4.1. Correlating OD-to-Bacteria Concentration

Correlating optical density (OD) to bacterial concentration is beneficial for the following reasons:

- Quick, non-destructive measurement: OD provides a fast, non-destructive way to estimate bacterial concentration, allowing real-time monitoring without sampling, which is useful when sample availability is limited.
- Cost-effective and accessible: OD measurements require only basic laboratory equipment, making this method both affordable and widely accessible for routine use in microbiology laboratories.
- High throughput for growth tracking: OD measurements enable frequent, high-throughput sampling to track growth phases (lag, exponential, stationary), essential for understanding bacterial dynamics and optimizing culture conditions.
- Standardized comparisons: establishing an OD-to-concentration correlation allows standardized measurements, simplifying comparisons across experiments, strains, and conditions.
- Supports downstream analysis: knowing bacterial concentration through OD is valuable for downstream processes, such as determining dosages in antimicrobial testing or scaling cultures for bioproduction.

Despite this, it is difficult to find complete tables showing the concordance between OD and bacterial concentration, even in the case of representative bacteria such as those belonging to the ESKAPEE group. This concordance undoubtedly depends on the production methods used, which can have an impact on the optical properties of the bacteria.

For this article, bacteria were grown according to the protocol used in industrial production. Relationships between OD and concentrations could be established whatever the measurement wavelength used. Differences may exist depending on the experimental conditions used in the laboratory, but with a constant protocol, the differences in slope between bacteria remain the same, providing a basis for comparison with ESKAPEE bacteria. In any case, this does not call into question the method of determining the measurement wavelength presented here, which applies whatever the experimental protocol for bacterial culture.

### 4.2. Common Reasons for Chosing a Measurement Wavelength and Situation with Bacteria

It is generally recognized that optical density (OD) measurements are commonly conducted between 600 nm and 700 nm, with some cases extending to longer wavelengths [7,9,10,11,12]. One answer often given during quick surveys is that the measurement wavelength chosen corresponds to the maximum absorption of suspensions. This contradicts the main reasons given in the literature for the high diffraction and low absorption of bacteria. However, this answer may apply to certain instances, such as eukaryotes (e.g., B-cells or T-cells, which absorb maximally around 550 nm and 650 nm, respectively [15,16]), but it does not fully account for all scenarios. For prokaryotes/bacteria, for instance, there is no absorption maximum within the visible spectrum (Figure 1).

The assumption that the OD-to-concentration (ODvC) relationship is linear across certain wavelengths has also emerged. However, as demonstrated in the R^2^ stability range (Figure 4), this assumption does not hold true for bacteria. Additionally, it is sometimes suggested that specific wavelengths are selected to minimize interference from culture medium [7]. However, transmittance and consequently OD measurement rely on a comparative analysis: the amount of light passing through a spectroscopy cuvette with culture medium versus an identical cuvette with only the culture medium. This approach effectively neutralizes both spectral characteristics of the light source and any absorbance contribution by the culture medium.

Other rationales have been proposed for choosing 600 nm, such as reduced UV-related harm to biological elements [17]. While this is accurate, it does not necessarily validate 600 nm as the optimal or exclusive measurement wavelength. Similarly, the assertion that scattering efficiency is highest at 600 nm is debatable, as scattering efficiency in the visible range tends to decrease gradually with 1/λ for micro-sized particles [13].

Originally, the choice of wavelengths used was mainly guided by the light sources available in the equipment used. Spectral analysis was not yet widely used for this type of application. The reason for a specific measurement wavelength most often put forward in the literature is that bacteria diffract light very efficiently, while their internal constituents do not absorb it [3,7]. However, the link between the chosen measurement wavelength and potential accuracy in terms of bacterial concentration has not been clearly demonstrated. The relevance of favoring diffraction over absorption would undoubtedly merit further investigations, made possible by the equipment currently available.

To summarize, a review of the scientific literature and an exploration of online resources reveal a range of measurement wavelengths but no clear, objective methods for determining the most suitable wavelengths for specific biological targets. This article seeks to offer some clarity on this matter.

### 4.3. Noise Level at High Concentrations and Short Wavelengths

Spectra, slopes, and R^2^ (Figure 1, Figure 3, and Figure 4, respectively) are noisy for high concentrations at short wavelengths, mainly due to the compact component used in our system, developed initially for in-line quality control of CAR T manufacturing without sampling in a closed system configuration. We did not consider methods based on the use of ultra-sensitive detectors such as photomultiplier tubes coupled to monochromators, which are commonly used in plate readers. Therefore, data corresponding to high concentrations were noisy at short wavelengths because the spectrometer we used was not sensitive enough to measure transmission below 1%, i.e., OD ≥ 2.

### 4.4. R^2^ Stabilization Ranges

Stabilized R^2^ values were greater than 0.9 for *E. coli*, *S. aureus*, *E. faecium*, and *E. cloacae*. Meanwhile, stabilization wavelengths were ≤400 nm for the same bacteria except *S. aureus*, where the stabilization wavelength is high: 460 nm.

High R^2^ and short stabilization wavelengths for *E. coli*, *E. faecium*, and *E. cloacae* may be due to the ease of cultivation and handling of these bacteria (see Section 4.5). This is consistent with the dispersions calculated in a previous paper on the absorption spectra shapes of ESKAPEE bacteria: these bacteria showed the lowest dispersion of the models describing the shapes of their absorption spectra [4]. Indeed, *E. faecium* and *E. cloacae* showed the shortest stabilization wavelengths (370 and 390 nm, respectively) and high R^2^ values. Considering also the dispersions mentioned above, it could be concluded that these bacteria could be good candidates as model bacteria for studies with bacteria, as *E. coli* is. Despite its stabilization wavelength of 460 nm, *S. aureus* could also be used as a model bacterium.

Conversely, *K. pneumoniae* showed a large stabilization wavelength and a low stabilized R^2^ value. According to the above hypothesis, this bacterium should not be considered as a model. The same applies to *A. baumannii* and *P. aeruginosa*, for which no stabilization was observed. Note that *K. pneumoniae* and *A. baumannii* showed the highest dispersion. Surprisingly, the R^2^ of *P. aeruginosa* did not stabilize while this bacterium showed a dispersion close to that of S. aureus. There is no explanation for this.

Intuitively, this implies that the measurement wavelengths for bacteria should be chosen above their stabilization wavelengths (except for *A. baumannii* and *P. aeruginosa*). The numerical results presented in this paper confirm this assumption.

### 4.5. Noise Calculated for OD = 0.5, Nature of the Extracted Noise, and SNR

The noise shown in Figure 6 is not due to electronic detection noise alone. If this were the case, the noise would be the same for all bacteria, and its minimum would always occur at the same wavelength. The noise is therefore dependent on the bacteria considered, which may seem surprising. In fact, the noise extracted corresponds to noise in the estimation of slopes by fitting. This noise is due to the fact that, from one measurement to another, bacterial suspensions differ slightly from an ideal suspension. Indeed, this was the source of the dispersions already shown in [4], which reflected how measured spectra differed from ideal theoretical spectra.

The extracted noise originates in the difficulties of manipulating bacteria (cultivation, representative sampling of cultures, and enumeration by cfu.mL^−^^1^ measurements).

With regard to the evolution of SNRs with measurement wavelength, we note that they all converge towards values leading to theoretical measurement accuracies of the order of 0.3%. These are indeed “maximum” theoretical accuracies, as the actual precision depends precisely on the difficulties, which are bacteria-dependent, of manipulating the latter.

Also, the SNR tended to increase again above 750 nm wavelength for *E. coli* and *E. faecium*. This is because the noise amplitude tended to stabilize above these wavelengths. Experiments were not performed above 850 nm because the spectral range of our spectrometer did not allow it. It could be assumed that higher SNRs could be achieved with these two bacteria for wavelengths in the near-infrared region. This could be especially interesting in experiments where very low bacterial concentrations are considered. Some plate readers cover the NIR wavelength up to 1000 nm (e.g., Infinite^®^ M Nano from Tecan [18]). Otherwise, the use of spectrophotometers would be required.

Conversely, the smaller the measurement wavelength, the greater the measurement dynamic (Figure 5). However, this high dynamic range is achieved at the cost of lower accuracy (Table 4). It is interesting to note that the optimum measurement wavelengths correspond to a spectral zone where not only accuracy is best, but also measurement dynamics are intermediate.

### 4.6. Concerning the Term Optical Density

Originally, the notion of OD comes from Beer-Lambert’s law, which measures the concentration of dissolved substances. In this law, solutions are considered homogeneous, and only light absorption is taken into account [19]. In the case of microorganism suspensions, the notion of a homogeneous solution is questionable. The literature insists that microorganisms of the size of bacteria diffract light rather than absorb it. However, absorption phenomena also exist. Whatever is not incident on the optical detector has either been absorbed or diffracted. In this case, the term absorbance spectrum or OD spectrum is somewhat inappropriate. The term extinction spectrum should be preferred, as it was used in the first experiments on the turbidity of microorganisms [3].

### 4.7. Extension to Other Organisms/Biological Elements and Ease of Implementation

In cases where absorbance spectra show well-identified maxima, the wavelength of measurement should be chosen at values where absorbance is at a maximum. For B lymphocytes, the absorbance maximum occurs around 640 nm [15]. For T lymphocytes, this maximum is around 530 nm [16]. For yeast such as C. albicans, it is located around 470 nm [15]. For breast cancer-derived exosomes, an absorption maximum is observed around 420 nm [20].

However, the method described here can be applied to any type of microorganism or biological element whose absorbance spectrum has no obvious characteristics that would allow fixing a particular measurement wavelength. This is the case for bacteria, but also for other biological elements of micrometric size for which diffraction is the predominant light-matter interaction process. For example, the method has been applied to *Lactococcus lactis*, for which the optimal wavelength is 638 nm. However, this paper being dedicated to the model group of ESKAPEE bacteria, *L. lactis* has not been included in the paper. Also, ESKAPEE bacteria represent a wide spectrum of bacteria characteristics (Gram+, Gram−, bacillus, and cocci), which indicates that the method is relevant to any bacteria type.

### 4.8. Comparison with Classical Techniques

Traditionally, detection methods rely on monitoring growth and measuring various changes, including alterations in gas composition within a sealed environment, as seen in CO_2_-based blood bacteria detection for sepsis diagnosis [21]; the generation of highly charged ionic metabolites that modify the electrical properties of the culture medium, measurable through impedance techniques [22,23]; the use of ATP as an indicator of microbial viability, though this requires filtration to differentiate bacterial ATP from other sources [24]; and thermal shifts detectable through microcalorimetry [25]. The effectiveness of these approaches varies depending on the type of microorganism. However, their reliance on bacterial growth prior to analysis restricts their application to cultivable species.

Alternative methods are also available. Label-free approaches such as cytometry [26] and lens-free imaging [27] have been explored. On the commercial front, technologies like the BactoBox for direct bacterial enumeration [28], automated seeders [29], and plate readers for optical density (OD)-based measurements [30] offer additional options.

Finally, the method presented here can be greatly simplified using plate readers and/or automatic seeders. For all ESKAPEE bacteria, a total of 255 spectra were recorded for this study. Modern plate readers can operate on plates with up to 1536 wells [30], so a single operation would have sufficed to carry out this work.

## 5. Conclusions

In this paper, an objective method for determining the optimum wavelength for measuring optical density has been proposed. It is based on an analysis of the signal-to-noise ratio of the curves representing the evolution of the measured concentration as a function of the measurement wavelength, whatever the optical density considered. As a preamble to this, a summary of absorbance-concentration relationships was provided, considering a measurement at 600 nm, the wavelength commonly used in the absence of a method for determining the ideal measurement wavelength.

The numerical procedure presented here can be applied to any type of suspension of biological element for which no measurement wavelength is directly identifiable. The spectral measurement capabilities and number of measurement wells accessible with current plate readers would also greatly simplify the method.

To the best of our knowledge, this is the first time that such a method for determining the measurement wavelength of optical densities measurement is proposed.

## Figures and Tables

**Table 1:** Number of exploited spectra and concentration range per species.

Bacteria	*Escherichia* *coli*	*Staphylococcus* *aureus*	*Klebsiella* *pneumoniae*	*Acinetobacter* *baumannii*	*Pseudomonas* *aeruginosa*	*Enterococcus* *faecium*	*Enterobacter* *cloacae*
**Spectra (n=)**	40	35	35	36	35	35	39
**Lowest concentration (bact.mL^−1^)**	1.69 × 10^6^	7.24 × 10^6^	4.26 × 10^6^	2.55 × 10^6^	5.20 × 10^6^	1.93 × 10^6^	1.61 × 10^6^
**Highest concentration (bact.mL^−1^)**	5.39 × 10^8^	1.21 × 10^9^	7.87 × 10^8^	6.35 × 10^8^	1.23 × 10^9^	2.64 × 10^8^	3.9 × 10^8^

**Table 2:** Slopes of the linear ODvC relationships and expected concentrations for OD = 0.5. Results for a measurement at 600 nm wavelength.

Bacteria	*Escherichia* *coli*	*Staphylococcus* *aureus*	*Klebsiella* *pneumoniae*	*Acinetobacter* *baumannii*	*Pseudomonas* *aeruginosa*	*Enterococcus* *faecium*	*Enterobacter* *cloacae*
**Slope @ 600 nm (mL.bact^−1^)**	2.40 × 10^−9^	1.04 × 10^−9^	2.04 × 10^−9^	2.18 × 10^−9^	9.40 × 10^−10^	4.19 × 10^−9^	2.73 × 10^−9^
**Conc. For OD_600_ = 0.5 (bact.mL^−1^)**	2.10 × 10^8^	4.80 × 10^8^	2.40 × 10^8^	2.29 × 10^8^	5.30 × 10^8^	1.19 × 10^8^	1.83 × 10^8^

**Table 3:** Stabilized R^2^. Starting stabilization wavelengths and R^2^ values.

Bacteria	*Escherichia* *coli*	*Staphylococcus* *aureus*	*Klebsiella* *pneumoniae*	*Acinetobacter* *baumannii*	*Pseudomonas* *aeruginosa*	*Enterococcus* *faecium*	*Enterobacter* *cloacae*
**Minimum λ (nm)**	400	460	530	>400	>400	370	390
**R^2^ value or range**	0.96	0.93	0.86	0.83–0.88	0.88–0.90	0.92	0.92

**Table 4:** Numerical values corresponding to SNR studies.

Bacteria	*Escherichia* *coli*	*Staphylococcus* *aureus*	*Klebsiella* *pneumoniae*	*Acinetobacter* *baumannii*	*Pseudomonas* *aeruginosa*	*Enterococcus* *faecium*	*Enterobacter* *cloacae*
**Min. noise amplitude** **(bact.mL^−1^)**	8.03 × 10^5^	2.02 × 10^6^	8.97 × 10^5^	1.03 × 10^6^	1.90 × 10^6^	4.00 × 10^5^	6.83 × 10^5^
**λ for min. noise ** **(nm)**	640	651	640	661	630	596	614
**Max. SNR** **(no units)**	309	293	320	290	324	299	288
**λ for max. SNR** **(nm)**	656	669	656	705	664	612	627
**Accuracy variation (%)**	6 → 0.32	2 → 0.34	1.7 → 0.31	6.5 → 0.34	3.6 → 0.31	2.2 → 0.33	4.6 → 0.35

## Data Availability

Research data are available on demand to the corresponding author.
